# Using Psychometric Network Analysis to Examine the Components of Spoken Word Recognition

**DOI:** 10.5334/joc.340

**Published:** 2024-01-10

**Authors:** Florian Hintz, James M. McQueen, Antje S. Meyer

**Affiliations:** 1 Philipps University of Marburg, Marburg, Germany; 2 Max Planck Institute for Psycholinguistics, Nijmegen, The Netherlands; 3 Radboud University, Nijmegen, The Netherlands

**Keywords:** individual differences, word recognition, network analysis

## Abstract

Using language requires access to domain-specific linguistic representations, but also draws on domain-general cognitive skills. A key issue in current psycholinguistics is to situate linguistic processing in the network of human cognitive abilities. Here, we focused on spoken word recognition and used an individual differences approach to examine the links of scores in word recognition tasks with scores on tasks capturing effects of linguistic experience, general processing speed, working memory, and non-verbal reasoning. 281 young native speakers of Dutch completed an extensive test battery assessing these cognitive skills. We used psychometric network analysis to map out the direct links between the scores, that is, the unique variance between pairs of scores, controlling for variance shared with the other scores. The analysis revealed direct links between word recognition skills and processing speed. We discuss the implications of these results and the potential of psychometric network analysis for studying language processing and its embedding in the broader cognitive system.

## Introduction

A comprehensive theory of the cognitive architecture that supports the human ability to use language – to speak, listen, read, write, gesture and sign – is one that fully specifies the mental representations and processes that support this ability. Psycholinguistic research has made considerable progress in specifying such an architecture (for a review see McQueen & Meyer, 2019). It is now broadly accepted that language processing not only requires access to domain-specific linguistic representations but also draws on domain-general skills, such as processing speed and working memory (e.g., Kidd et al., 2018). This view fits well with the broader framework of process overlap theory on human intelligence (Kovacs & Conway, 2016), which assumes that cognitive tasks tap both domain-general and domain-specific processes and that individuals’ general cognitive ability (g) emerges as the overlap of domain-general and domain-specific processes tapped across different cognitive tasks.

Though there is broad agreement in the field that the language system overlaps with other cognitive systems, the extent to which specific components of the language system intersect with specific components of other cognitive systems is largely unknown, and so the general architecture of the cognitive system underlying language use is still seriously underspecified. As a step towards addressing this broad issue, we focussed on spoken word recognition and asked how much word recognition draws on domain-general and domain-specific skills. To answer this question, we adopted an individual-differences approach and tested a large sample of participants on tests tapping word recognition, linguistic knowledge, and domain-general skills. We then used psychometric network analysis (e.g., Borsboom & Cramer, 2013; Goring et al., 2021; Schmank et al., 2019) to compute and visualise the unique relationships between test scores. The present paper has two aims: to report novel findings about the relationships between the word recognition system and other components of the cognitive system, and to illustrate the use of network analysis in individual-differences work in psycholinguistics.

In the remainder of this Introduction, we first sketch a working model of auditory word recognition and its embedding in the broader cognitive system. We lay out how individual-differences studies can contribute to an understanding of the architecture of the cognitive system. We then provide an overview of our study, relevant background information about network analysis, which is the statistical tool we used, and finally explain the predictions for the network analysis.

### A working model of spoken word recognition

The present work concerns the recognition of spoken words presented in isolation. Most models (e.g., Magnuson et al., 2020; McClelland & Elman, 1986; Norris & McQueen, 2008) assume that auditory word recognition proceeds in three temporally overlapping stages: Listeners map the auditory information onto sublexical units, select the best-fitting word and then select the word meaning (Eisner & McQueen, 2018, for review). Word recognition is assumed to be incremental in that auditory input is processed immediately, rather than being buffered. Moreover, word recognition is assumed to be competitive in that activation at sublexical levels is assumed to partially activate multiple lexical candidates, which compete for recognition (e.g., Allopenna et al., 1998).

Though auditory word recognition is driven by the speech signal and requires access to stored lexical and conceptual representations, it is affected by domain-general processes. Previous behavioural and neurobiological studies revealed critical roles for working memory and processing speed during spoken word recognition (e.g., Hadar et al., 2016; Hintz et al., 2020a; Klimovich-Gray & Bozic, 2019; Nitsan et al., 2019). However, little is known about exactly where and how domain-specific and domain-general processes interact in spoken word recognition. Our research aims to address this issue.

### Assessment of individual differences to study the cognitive network

Much of the work on the embedding of language processing in the broader cognitive system has used an individual-differences approach, where participants are asked to perform a language task as well as tests measuring the relevant domain-general processing skills. The patterns of variability across individuals in their performance on those tests are examined, and correlations point to the involvement of shared cognitive processes. In many of these studies, participants carry out a single language task (e.g., auditory lexical decision to gauge word recognition, Goldinger, 1996) and one or more tests of domain-general skills (e.g., forward and backward versions of the Digit Span test to gauge working memory, Wechsler, 2004). Researchers choose such designs because they aim to test specific hypotheses, for instance whether working memory affects word recognition accuracy or speed (see Carpenter & Just, 2013, for a review on the role of working memory in language processing).

However, the interpretation of the correlations within a processing model is challenging for a number of reasons, all related to the fact that complex cognitive tasks, such as spoken word recognition and tasks primarily tapping into working memory (e.g., Digit Span), draw on multiple cognitive skills and so the functional of a correlation cannot be determined. For instance, a correlation between working memory and lexical decision performance may arise because lexical decision requires working memory, or because both tasks require rapid processing of incoming auditory information, that is, both tasks involve another domain-general skill, namely processing speed.

A way to address this issue is to assess participants’ cognitive skills as broadly as possible (for discussion of this general approach see Miyake et al., 2000). For instance, instead of using a single task to assess word recognition, one would use several tasks, designed to assess word recognition in different ways. Furthermore, in addition to measuring the skills of primary interest (e.g., word recognition and working memory), one would measure skills likely to be correlated with those target skills, for instance vocabulary size and processing speed. Different statistical tools can then be used to examine the entire matrix of correlations, to explore which test scores cluster together and presumably measure the same skill, and which unique links emerge between scores when variance shared with other scores is controlled for. In the present study, we used such a broad assessment of cognitive skills and applied network analysis to analyse the dataset.

### Overview of the study

We used an individual-differences approach with a large sample of adult native speakers of Dutch to study the embedding of word recognition in the broader cognitive system. We went beyond most studies in the field by using a broader assessment of word recognition and a more comprehensive test battery of linguistic and domain-general skills. We were particularly interested in the impact of processing speed on auditory word recognition and explored whether we could pinpoint where (i.e., at which processing stage) during word recognition processing speed was most important. To that end, we used three word recognition tasks, primarily targeting sublexical, word form and meaning level processing, respectively. We assessed processing speed in a battery of five tasks. In addition, we assessed working memory, non-verbal reasoning and, in a battery of tasks, effects of linguistic experience. As noted, we used network analysis to determine and visualise the unique links between tests. In this section, we provide an overview of the tasks. More detail is given in the Methods section below.

To be able to draw a fine-grained picture of the involvement of domain-general processes in word recognition, we assessed word recognition in three tasks. Consistent with earlier work, we chose rhyme judgment for non-words to target sublexical, phonological processing (McQueen, 1993); we chose lexical decision to target word-form processing (Goldinger, 1996); and we chose semantic categorisation to target word-meaning processing (Collins & Loftus, 1975).

Evidently, no test is a pure measure of only one type of processing. However, the tests vary in the kinds of representations that minimally need to be accessed to perform them. In rhyme judgement for non-words, word form and word meaning access may occur, but only access to sublexical phonological representations is strictly necessary. For lexical decision, word meaning access may occur, but only access to sublexical and word-form representations is required. Finally, for semantic categorisation, access to all three kinds of representations is needed. Thus, semantic categorisation may be seen as the most complex task, requiring processing at all three representational levels, and rhyme judgment may be seen as the least complex task, requiring processing only at the phonological level. As will be further explained below, we examined whether the three tasks differed in their links to scores of tests tapping domain-general and linguistics skills.

Concerning the impact of domain-general processes, we were specifically interested in determining the links between the individual word recognition tasks and non-verbal processing speed tasks (for related work, see Hintz et al., 2020a). We assessed non-linguistic processing speed in five tasks, all requiring speeded button responses. Since the three word recognition tasks also involved this response type, it was plausible to assume a link with non-verbal processing speed, which would reflect variability across participants in carrying out speeded manual responses. An important question was whether processing speed would show links to word recognition when variability in conducting a speeded manual task was accounted for, that is, whether processing speed was involved in processing at sublexical, lexical and semantic levels of representation.

A related question was whether word recognition would show tighter links to non-verbal processing speed as task complexity increased. Compared to rhyme judgment for non-words, semantic categorisation may be more tightly linked to non-verbal processing speed, as non-verbal processing speed may contribute to the speed with which activation cascades through the word recognition system (i.e., from phonological to word form and to semantic levels of representations). Lexical decision and semantic categorisation require the evaluation of alternative lexical hypotheses in step with the changing input signal. Better non-verbal processing speed skills may also benefit this process. An alternative hypothesis is that there is little variability pertaining to the speed with which activation cascades from phonological through semantic levels of representation across adult individuals. This would speak for a highly automatised process, making the recruitment of domain-general processing skills unnecessary.

In addition to word recognition and processing speed, we assessed the participants’ working memory, using two tests, and their non-verbal reasoning ability. These tests were included because earlier studies have shown that working and/or non-verbal reasoning ability are correlated with processing speed (Conway et al., 2002; Salthouse, 1996), and we aimed to investigate the link between processing speed and word recognition after controlling for such influences. In addition, the rhyme judgment and semantic categorisation tests required participants to keep linguistic information in working memory for a short period of time. In the rhyme judgment test, participants first heard a cue non-word and were instructed to judge whether the cue and the subsequent target non-word rhymed. In the semantic categorisation test, participants were given a semantic category before the task and were instructed to indicate for each of a series of test words whether or not it belonged to that category. In both cases, working memory skills could influence test performance.

Finally, we included a set of tests measuring linguistic experience. These were tests measuring receptive and productive vocabulary size, exposure to literary texts, spelling skills, knowledge of idiomatic expressions and prescriptive grammar. Our primary motivation for including these tests was again to rule out the influences of linguistic knowledge when assessing the influence of processing speed on lexical decision. This was important because earlier studies have shown that measures of linguistic experience affect lexical decision times (e.g., Diependaele et al., 2013). Diependaele and colleagues argued that individuals with more linguistic experience, for which they used a test of receptive vocabulary size (LexTALE, Lemhöfer & Broersma, 2012) as a proxy, have more entrenched or sharper lexical representations, which facilitate word recognition. While it is plausible that individuals with more linguistic experience have larger vocabularies than individuals with limited experience, a test of receptive vocabulary alone is insufficient to fully capture the breadth of one’s linguistic experience. We therefore included these five additional tests to increase the scope of the linguistic experience measures.

In sum, participants were tested on a battery of 19 tasks. After pre-processing we correlated the test scores and performed a network analysis to determine and represent the links of the word recognition tasks to the remaining tasks.

### Psychometric network analysis

A suitable analytical tool for addressing our research questions, which deals with multivariate relationships among test scores, is psychometric network analysis (Epskamp & Fried, 2018; Epskamp et al., 2018). Psychometric network analysis conceptualizes and visualises complex psychological attributes or behaviours as interconnected networks. In these networks, the observations (e.g., test scores) are referred to as nodes and the connections between pairs of nodes are referred to as links or edges. A link connecting a pair of nodes reflects the direct relationship between these nodes, controlling for variance that is shared with the rest of the network. A regularization algorithm removes spurious correlations. Similar to latent variable modelling, psychometric network analysis is an exploratory estimation technique that can be used to assess the underlying interconnectedness of observed data. One of the strengths of psychometric network analysis is that it offers an explanation for the *positive manifold* (Spearman, 1904), which is the observation that participants who score high on one cognitive test are likely to score high on other cognitive tests as well. Whereas latent variable modelling approaches assume that performance in individual tasks is driven by some higher-order latent factor (i.e., a common factor extracted from the shared covariance among observed variables), psychometric network analysis does not assume the presence of latent factors but represents the unique links between scores. Well-connected clusters of nodes can then be interpreted as representing a common construct. This analytical approach fits well with recent theories of human cognitive functioning, such as Process Overlap Theory (POT) by Kovacs and Conway (2016; see van der Maas et al., 2006, for a related account), which view the cognitive system as a set of overlapping skills implemented in overlapping neural circuits.

### Predictions

We used network analysis to explore the links between the scores participants obtained in our test battery and assessed the hypotheses about the relationship between word recognition, processing speed, working memory and linguistic experience introduced above.

1. We hypothesised that scores (i.e., nodes) representing the same psychological construct (i.e., spoken word recognition, linguistic experience, processing speed, and working memory) cluster together in the network. This is because the overlap in processes tapped by tests measuring the same construct should be large, resulting in strong links between the scores.
2. Concerning the links between scores of spoken word recognition, we hypothesised a stronger link between lexical decision and semantic categorisation than between rhyme judgment and semantic categorisation due to the larger overlap in processing at sublexical, lexical and semantic levels of representation.
3. We hypothesised links between the scores of spoken word recognition and those of non-verbal processing speed (cf. Hintz et al., 2020a). Importantly, since the tests of spoken word recognition and the tests of processing speed *all* involved button presses under time pressure, variance associated with carrying out a speeded manual response was ‘accounted for’ such that any link between a word recognition and processing speed test reflects shared variance *beyond* that involved in speeded manual responding. An open question was whether the number or strengths of links between word recognition and processing speed scores varied as a function of the complexity of the word recognition task.
4. We hypothesised that spoken word recognition would show links to linguistic experience. In line with the results by Diependaele et al. (2013), lexical decision and semantic categorisation tests should show more and/or stronger links to linguistic experience than rhyme judgement for non-words as, arguably, enhanced exposure to language in typically developed younger adults should primarily affect processing at lexical and semantic levels.
5. Finally, as explained above, the rhyme judgment and semantic categorisation tasks involved remembering a cue non-word and a semantic category, respectively. The lexical decision task does not have this memory component. We therefore predicted links between the former two spoken word recognition tasks and working memory but not between lexical decision and working memory.

## Methods

### Dataset and participants

The dataset we used for the present analysis is a merger of data from two studies (set A and set B hereafter) that were run in the context of a research program on individual differences in language skills carried out at the Max Planck Institute for Psycholinguistics. Between 2017 and 2022, we developed a new behavioural test battery consisting of 31 tests that measure individual differences in linguistic knowledge, general cognitive skills and in linguistic processing skills. Set A, with 112 participants, stems from a large pilot study we ran in 2019. The data are available to the research community (Hintz et al., 2020b). Between 2020 and 2022, we collected data from more participants on these tasks (Hintz et al., in prep.). While 579 of them completed the tasks via the internet, 169 participants completed them at the Max Planck Institute for Psycholinguistics, as the participants in our earlier study had done (Hintz et al., 2020b). These 169 participants constituted Set B.

Dataset A comprised the results obtained from 112 participants. Seventy-three of these participants were female and 39 were male; the mean age was 22.29 years (SD = 2.80, range = 18–29). Dataset B stemmed from 169 participants. Eighty-five of these participants were female, 82 were male, and two identified as other; the mean age was 22.38 years (SD = 2.76, range = 18–30). At the time of testing, most participants in both sets were enrolled in a study program either at a university or a university of applied sciences. All participants were native speakers of Dutch. All participants gave written informed consent to take part in the study and were paid for participation. Permission to conduct the study had been provided by the Ethics Board of the Social Sciences Faculty of Radboud University.

The total sample size of 281 participants is well in line with the simulation results by Constantin and Cramer (2022), who found that sample sizes ranging from 250 to 350 are generally enough to achieve moderate sensitivity, high specificity, and high edge weight correlations when psychometric networks are sparse and consist of 20 nodes or less, as was the case in the present analysis.

### General procedure

Participants were tested in a quiet test room at the Max Planck Institute for Psycholinguistics. They were tested in groups of up to eight individuals at the same time. Each participant was seated at a desk with an experimental laptop, a computer mouse and a custom-made button box (two buttons) in front of them. Experimental laptops were Hewlett Packard ‘ProBooks 640G1’ with 14-inch screens, running Windows 7, optimized for experimentation. Participants were seated in a semicircle around the room facing the wall, with approximately 1 m – 1.5 m space between them. Noise cancelling divider walls (height 1.80 m, width 1 m) were placed between participant desks and the walls in front of them were covered with curtains to absorb as much noise as possible. Beyerdynamic DT790 headsets were used for the presentation of the auditory stimuli.

The tests were either implemented in Presentation© (version 20.0, www.neurobs.com) and run ‘locally’ on the laptops or were implemented as a web application in ‘Frinex’ (Framework for Interactive Experiments, an environment developed by the technical group at the Max Planck Institute for Psycholinguistics, Monen et al., in prep.) and run online in the laptops’ web browser (Chrome, version 75.0.3770.142). Specifically, all tests where exact timing was critical (rhyme judgment, auditory lexical decision, semantic categorisation, auditory simple RT test, auditory choice RT test, letter comparison, visual simple RT test, visual choice RT test) were run in Presentation, while the remaining tests were implemented in Frinex (Digit Span, Corsi block, Raven’s Advanced Progressive Matrices, Peabody Picture Vocabulary Test, antonym production, spelling test, Dutch Author Recognition Test, idiom recognition, prescriptive grammar). As Frinex has been developed only recently, we did not have reliable data concerning its timing precision (i.e., time stamping of auditory, visual and response events) and therefore decided to rely on the Presentation software for the chronometric tests.

Audio recordings for the respective tests were made in a soundproof booth using a Sennheiser microphone sampling at a frequency of 44 kHz (16-bit resolution). Different native speakers of Dutch produced the stimuli for each test. All stimuli were produced at a normal pace with neutral intonation. Audacity® (version 2.0.6, Audacity Team, 2014) was used to cut the recordings into individual audio (.wav) files.

The tests analysed in the present study represent a subset of the tests that both participant samples completed. In both groups, completing all tests took approximately four hours. In Hintz et al. (2020b, Dataset A of the current study), participants completed all of them on the same day (two sessions of one hour each in the morning and two sessions of one hour in the afternoon). In our later study (Dataset B of the current study), the participants completed them on two different test days (two hours of testing on each day). The order of tests was the same for all participants in both groups, as was the order of trials within each test. The participants in Hintz et al. (2020b, Dataset A of the current study) additionally completed all the tests a second time, four weeks after the first test day, to assess test-retest reliability of the tests. The majority of tests had good to very good retest reliability (see Online-Table 2 in Hintz et al., 2020b, for details). For the present analysis, data from the first test day were taken.

### Test descriptions

Below we summarise the test materials and procedure for each of the tests included in the present analysis. If not stated otherwise, both groups of participants carried out the same versions of the tests. Detailed descriptions of the tests can be found in Hintz et al. (2020b, in prep.).

#### Auditory word recognition tasks

Participants carried out three auditory word recognition tasks, a rhyme judgment task for non-words, an auditory lexical decision task, and a semantic categorisation task.

In the rhyme judgment task, participants were asked to judge as quickly as possible whether two successively presented non-words rhymed. The test consisted of four practice trials, followed by 40 test trials, of which 24 trials were rhyming trials (rhyme, e.g., ‘staum’ – ‘graum’), eight were foil trials (no rhyme, but non-words shared the same vowel nucleus, e.g., ‘floes’ – ‘broel’) and eight were unrelated trials (e.g., ‘treis’ – ‘breuf’). Target, foil and unrelated trials were presented in a pseudorandom order, such that there were never more than three subsequent target trials. The programme Wuggy (Keuleers & Brysbaert, 2010) was used to transform Dutch words into non-words without violating phonotactic constraints.

At the beginning of each trial, a fixation cross was displayed on the screen for 500 ms, after which the two non-words were presented with an inter-stimulus interval of 500 ms. Participants were instructed to indicate as fast as possible whether the two non-words rhymed or not, by pushing the correct button on the button box. The inter-trial interval was 2000 ms. Average response time, measured from the onset of the second non-word and based on correct responses to rhyming trials, was taken as the performance indicator.

In the auditory lexical decision task, participants heard single words and non-words, and were instructed to determine whether each one was or was not an existing Dutch word. Participants carried out three practice trials (two non-words and one word) followed by 120 test trials, of which 60 were words and 60 were non-words. The words were selected from the Subtlex database (Keuleers et al., 2010), and were likely to be known to all participants, as established using prevalence norms (Keuleers et al., 2015), but varied substantially in word frequency (M = 3.65, SD = 0.85, range = 2.04–5.66, as recommended by Van Heuven et al., 2014); we transformed the raw frequencies of occurrence into Zipfian frequencies).

For each Dutch word, a non-word was constructed using Wuggy (Keuleers & Brysbaert, 2010) by substituting two phonemes without violating any phonotactic constraints of Dutch. One additional word and two non-words were used as practice items. Words and non-words were presented in a pseudorandom order, such that participants never heard more than three words or non-words in a row.

Each trial started with the presentation of a fixation cross for 300 ms, followed by the auditory stimulus. Participants were instructed to listen carefully and to decide as quickly as possible whether or not they heard an existing Dutch word by pressing the corresponding button on the button box. Their response terminated the trial. The inter-trial interval was one second and the response time was the time interval between the spoken word onset and the button press. The performance indicator was the average response latency, measured from spoken word onset, on correct responses to words.

In the semantic categorisation task, participants were asked to judge whether the presented spoken words belonged to a specific category, which was provided ahead of time. The test was divided into two parts with one semantic category per part (professions, e.g., ‘bakker’, *baker* and vehicles, e.g. ‘politieauto’, *police car*). Each part of the test consisted of four practice trials followed by 32 test trials. Of these test trials, 20 were target items (i.e., words that belonged to the respective category) and twelve were unrelated distractors. Targets and distractors were presented in pseudorandom order, such that participants never heard more than three subsequent target trials. Targets and distractors in each category were matched on number of letters, number of phonemes, number of syllables, frequency, prevalence, and phonological neighbourhood density.

At the start of each trial, a fixation cross was presented for 500 ms after which participants heard the stimulus word. Their task was to indicate as fast as possible whether the word they heard referred to a member of the semantic category provided beforehand, by pushing the corresponding key on the button box. The inter-trial interval was 2000 ms. Participants’ average response time, measured from spoken word onset, based on correct responses to semantic category members, was taken as the performance indicator.

Note that the test set for the category ‘professions’ was slightly different for the two participants groups, as we updated the test and included more names of professions marked for female gender (e.g., lerares, “female teacher”) in the later study. Apart from these changes, the materials and procedure were the same in both studies. For both datasets, we calculated internal consistency based on ICC3 in R’s ‘psych’ package. These analyses showed that internal consistency in both datasets was very high and very similar (earlier study: .94, later study: .95).

#### Tests of processing speed

As in Hintz et al. (2020a), we used five tests to tap response speed to auditory and visual stimuli. In all tests, participants’ task was to respond as fast as possible to the onset of a stimulus. In the auditory simple reaction time test (RT), participants saw a fixation cross in the centre of the screen. After an interval varying between one and three seconds, a sine tone (550 Hz, 400 ms) was played to which participants should respond as quickly as possible. In its visual counterpart, participants responded to the appearance of a line drawing of a triangle (200 × 200 pixels, black contours). Each test consisted of 20 test trials. In the auditory choice reaction time test, participants responded as quickly as possible to each of two auditory stimuli (a low or high sine tone, 300 and 800 Hz, respectively, both 400 ms), presented in pseudo-random order, by pressing one of two buttons (left = low tone; right = high tone). In its visual counterpart, the tones were replaced with a line drawing of either a star or a circle (black contours, 200 × 200 pixels). Each choice reaction time test consisted of 40 test trials. Finally, we included a digitized version of the letter comparison test (Huettig & Janse, 2016; Salthouse, 1996), where participants were presented with two strings of letters and were asked to judge whether these were identical, using the appropriate button. The first test block featured three-letter-strings, the second block featured six-letter-strings (48 test trials in total). In all five tests, average response speed (for correct responses, in case of the choice tests), served as performance indicator.

#### Tests of working memory

We used a computerised version of the Digit Span test, adapted from Wechsler (2004), to assess auditory working memory and a computerized version of the Corsi block test (Berch et al., 1998; Chu et al., 2014) to assess visual working memory. At the beginning of each trial of the Digit Span test, a fixation cross appeared in the centre of the screen. After 2000 ms, the playback of a sequence of spoken digits was initiated while the fixation cross remained in view. Digits were presented at approximately 500 ms intervals. Following auditory playback, a response field appeared at the bottom of the screen and participants were requested to type out the digits in the order they were encountered (forward version) or in the reversed order (backward version). The first two trials of each version featured a two-digit sequence; these were considered practice trials. When at least one of two consecutive trials of the same length was recalled correctly, the sequence was extended by one digit. The test ended when two consecutive trials for a sequence length were responded to incorrectly or when participants reached the end of the test (nine digits in the forward version, eight digits in the backward version). Separate performance indicators were obtained for forward and backward versions, operationalized as the sum of correct responses per version.

In the Corsi block test, participants were presented with nine squares, which were randomly distributed across the screen. Different squares lit up successively at a rate of one square per second. At the end of a sequence, a green frame appeared around the display, prompting participants for a response. The participants were instructed to repeat the sequence by clicking on the respective squares, either by forward repetition or backward reproduction. When clicking on the squares, they briefly lit up in black for 200 ms and then turned blank again. After having reproduced the sequence in forward or backward fashion, participants clicked on a button at the bottom of the screen to proceed to the next trial. They were familiarized with the test, completing two practice trials of two-square sequences. The first experimental trial featured a sequence length of three squares. The sequence length was extended by one square when at least one of two consecutive trials was recalled correctly. The test ended when two consecutive trials for a given sequence length were responded to incorrectly or when participants reached the end of the test (sequence of nine blocks in both versions). The performance indicator was the sum of correct responses on experimental trials in forward and backward versions, respectively (Kessels et al., 2008).

#### Test of non-verbal reasoning

We included a computerized version of Ravens’ Advanced Progressive Matrices (APM, Raven, Raven, & Court, 1998), as a standardized test to tap non-verbal reasoning. On each trial, participants indicated which of eight possible shapes completed a matrix of geometric patterns. They selected the shape by clicking on it. Items could be skipped and were shown again at the end of the test. When participants did not know the answer to a skipped item, they could click on an ‘I don’t know’ button. The test consisted of 36 test items, increasing in difficulty. Participants had 20 minutes to complete the test items. The performance indicator was the proportion of correctly answered test items.

#### Tests of linguistic experience

We administered six tests of linguistic experience, the standardized Peabody Vocabulary Test, tests of antonym production, idiom recognition, and spelling, the Dutch Author Recognition Test, and a test of prescriptive grammar.

As a standardized test of receptive vocabulary size, we included a digitized version of the Dutch Peabody Picture Vocabulary Test (PPVT, Dunn & Dunn, 1997; Schlichting, 2005). On each trial, participants first previewed four numbered line drawings on their screen, heard a probe word, and indicated which of the four pictures best corresponded to the meaning of the spoken word by clicking on it. Participants could listen to the probe word as often as they wanted but had to listen to it at least once before a response was recorded. The test had 17 blocks, each consisting of twelve items of roughly the same difficulty. The test started at block 13 (normed entry block for participants aged between 18 and 35). Based on their performance (four or fewer errors in block 13), participants’ next block was either more difficult (block 14) or easier (block 12) than the entry block. The test terminated when more than eight errors were made within a block that was not the starting block or when a participant reached the last item of the test. Performance indicator was the difference between the item number of the last test word and the number of errors participants made.

An open-ended, untimed antonym production test was included to tap productive vocabulary. It was an adapted version of the test developed by Mainz et al. (2017) at the Max Planck Institute for Psycholinguistics. Participants were provided with a word cue and were instructed to produce its antonym (e.g., cue: hot, antonym: cold). The test consisted of 28 trials (3 practice and 25 experimental trials). Before each trial, participants saw a fixation cross for 500 ms, after which the cue word was presented (in written form and once in spoken form). Participants provided a spoken response, and their answer was recorded. They clicked on a button on the screen to advance to the next trial. The cue words varied in word frequency (Keuleers et al., 2010, M = 3.74, SD = 0.75, range = 1.70 – 5.00) and prevalence (Keuleers et al., 2015; M = 0.98, SD = 0.04, range = 0.85 – 1.00) and thus in how easily an antonym could be retrieved. Accuracy was coded offline by native Dutch research assistants at the Max Planck Institute for Psycholinguistics using a pre-defined set of responses. In case of an incorrect response that was semantically similar to the expected response, we checked on a case-by-case basis, using semantic similarity norms (Mandera, Keuleers & Brysbaert, 2017), whether this word could be accepted as a correct response. These cases were discussed until mutual agreement among all coders was reached. The performance indicator was the proportion of correct experimental trials.

We changed four items, which displayed poor agreement in the participants’ answers, from the earlier to the later version of this test. Internal consistency was comparable, if below accepted standards, across both versions (earlier: 0.59, later: 0.44).

Participants’ knowledge of Dutch idiomatic expressions was tested using an idiom recognition test. On each trial, the participants were presented with a Dutch idiom, such as ‘tussen de regels doorlezen’ (to read between the lines) and a set of four candidate meanings. They had to select the correct meaning among the set of four candidates. We selected a subset of ten items from the normative database described by Hubers et al. (2019). Specifically, to vary the item difficulty in our test, we selected items that ranged between 1.35 and 4.39 on the familiarity rating dimension (1–5 scale) and between 0.15 and 1 in meaning recognition accuracy as piloted by Hubers et al. (2019). An idiom was presented at the top of the screen and four meaning candidates were shown underneath in four quadrants of the screen. The position of the target meaning was varied across trials. Both the idiom and the four candidate meanings could be listened to (by mouse-clicking on a loudspeaker icon) to account for the possibility that some idioms are predominantly encountered in the spoken modality and to reduce the influence of reading skill on recognition performance. The performance indicator was the proportion of correctly recognised idioms.

Spelling skills were assessed in a test designed in our lab. The test consisted of a list of 60 Dutch words whose spelling adult language users often find difficult. These concern for example the correct use of diaeresis (i.e., ‘bacteriën’, bacteria), the use of double consonants in plural forms (‘slimmeriken’, wise guys), and use of ei/ij (diphthong [ɛi], i.e. ‘allerlei’, all kinds). Participants were presented with a list of 60 words, in pseudo-random order, divided into three columns of 20 words each. Half of the test words was spelled incorrectly. The ratio of correctly and incorrectly spelled words was not known to the participants. Participants were instructed to use their mouse to tick the boxes next to words they thought were spelled incorrectly. The participant’s performance indicator was the proportion correctly categorised misspelled words minus the proportion incorrectly selected words that were spelled correctly.

We included a digital version of the Dutch Author Recognition Test (Brysbaert et al., 2020). Participants were presented with a written list of 132 names, shown in three columns of 44 words each. They had to indicate which of the listed persons were authors (e.g., Roald Dahl, Nicci French). Ninety of the listed persons were authors and 42 were non-author foils. Authors and non-authors were listed in pseudo-random order and the ratio of authors/non-authors was not known to participants. The performance indicator was the proportion of correctly identified authors minus the proportion non-authors wrongly selected.

As for the semantic categorisation and antonym production tests, we updated some items (n = 25) of this test after our earlier study. As for the semantic categorisation test, our analysis of internal consistency (here based on Cronachbach’s Alpha) for both versions revealed high internal consistency in and high similarity between both versions (earlier study: .92, later study: .93).

To assess participants’ knowledge of Dutch prescriptive grammar, a recently developed grammaticality judgment test (Favier & Huettig, 2021) was used. Participants heard spoken sentences and indicated for each of them whether they thought it was a correct Dutch sentence. The sentences featured five grammatical categories (eight trials per category, 50% correct), which adult native speakers of Dutch often find difficult to use correctly: personal pronouns (‘ze’, they vs. ‘hun’, their; ‘ik’, I vs. ‘mij’, me), comparatives (‘als’, as vs. ‘dan’, than), relative pronouns (‘die’, this vs. ‘dat’, that) and participle formation of complex verbs (e.g., ‘stofzuigen’, to vacuum). Stimuli were recorded in a soundproof booth. Average sentence duration was 4344 ms (SD = 653, range = 3056 – 5901). Each trial started with the presentation of a fixation cross, which coincided with the playback of the spoken sentence. The fixation cross remained in view for the duration of the sentence. Each sentence was presented only once. Participants could respond during or after the presentation of the sentence by mouse-clicking on the appropriate button on the screen (labelled ‘correct’, right-hand position and ‘incorrect’, left-hand position). The mouse-click terminated the trial. The inter-trial interval was 500 ms. The performance indicator was the proportion of correct responses.

### Data pre-processing

For the tests with RT as dependent variable, incorrect responses (on tests with a choice component) were excluded. We then inspected the overall distribution of the data, identified mean, minimum and maximum in each distribution and decided – based on these values and our previous work (Hintz et al., 2020a) – for each test on specific thresholds for excluding extreme values. For rhyme judgment, auditory lexical decision, semantic categorisation, and letter comparison, the upper and lower thresholds were 300 ms and 3000 ms, respectively; for auditory and visual simple RT tests they were 100 ms and 1000 ms; and for auditory and visual choice RT tests they were 200 ms and 2000 ms. Across all RT-based tests, less than 2% of the data were excluded during pre-processing. Finally, RTs were log-transformed and inverted (i.e., multiplied by –1) such that for all 19 scores larger values reflect better performance. Performance indicators for spoken word recognition and processing speed tests were calculated for participants who retained minimally 80% of trials in the critical condition.

## Results and Discussion

Table 1 shows the descriptive statistics for all 19 tests. As can be seen, except for idiom recognition, antonym production and Corsi block (forward) tests, the internal consistency was good to excellent for all tests. Similarly, the score distribution across participants was normal as suggested by the skewness and kurtosis values: Skewness values were all between +/-1. Except for one measure (auditory choice RT), all kurtosis values were between +/-1.5.

**Table 1:** Descriptive statistics for the 19 tests.


	RJ	ALDT	SC	ASRT	ACRT	LC	VSRT	VCRT	DSF	DSB	CBF	CBB	PPVT	AP	IR	DART	SP	PG	RPM

Valid	272	279	278	280	276	271	281	279	281	278	277	276	281	280	281	278	281	281	278

Missing	9	2	3	1	5	10	0	2	0	3	4	5	0	1	0	3	0	0	3

Mean	–2.89	–2.94	–2.92	–2.32	–2.58	–2.99	–2.37	–2.61	8.32	7.04	8.31	7.59	174.93	0.77	0.77	0.23	0.57	0.69	0.58

SD	0.08	0.05	0.06	0.07	0.09	0.07	0.05	0.06	2.11	2.12	1.71	2.05	11.08	0.09	0.12	0.14	0.17	0.11	0.16

Skewness	–0.47	–0.61	–0.72	–0.94	–0.91	–0.07	–0.69	–0.78	0.28	0.10	0.07	0.13	–0.74	–0.55	–0.44	0.95	–0.36	0.02	–0.37

Kurtosis	0.02	0.40	1.08	2.59	0.95	–0.14	0.60	0.99	–0.62	–0.47	0.14	0.29	0.63	0.32	0.21	1.34	–0.32	–0.34	–0.07

Minimum	–3.14	–3.10	–3.17	–2.65	–2.92	–3.19	–2.55	–2.85	4.00	1.00	3	2	136	0.48	0.40	–0.03	0.10	0.40	0.14

Maximum	–2.72	–2.84	–2.80	–2.16	–2.42	–2.81	–2.25	–2.47	14	12	13	14	198	0.96	1.00	0.80	0.93	1.00	0.94

IC	0.94^a^	0.96^a^	0.94^a^ 0.95^a^	0.92^a^	0.96^a^	0.96^a^	0.87^a^	0.95^a^	0.72^b^	0.72^b^	0.59^b^	0.69^b^	0.91^b^	0.59^b^ 0.44^b^	0.25^b^	0.92^b^ 0.93^b^	0.78^b^	0.69^b^	0.85^b^


*Note*: RJ = Rhyme judgment, ALDT = Auditory lexical decision test, SC = Semantic categorisation, ASRT = Auditory simple reaction time, ACRT = Auditory choice reaction time, LC = Letter comparison, VSRT = Visual simple reaction time, VCRT = Visual choice reaction time, DSF = Digit Span forward, DSB = Digit Span backward, CBF = Corsi block forward, CBB = Corsi block backward, PPVT = Peabody Picture Vocabulary Test, AP = Antonym production, IR = Idiom recognition, DART = Dutch Author Recognition Test, SP = Spelling test, PG = Prescriptive grammar test, RPM = Raven’s Progressive Matrices, IC = Internal consistency. ^a^ICC3k calculated using the ICC() function of the psych R package. ^b^Cronbach’s Alpha, calculated using the cronbach.alpha() function of the ltm R package.

**Figure 1:**
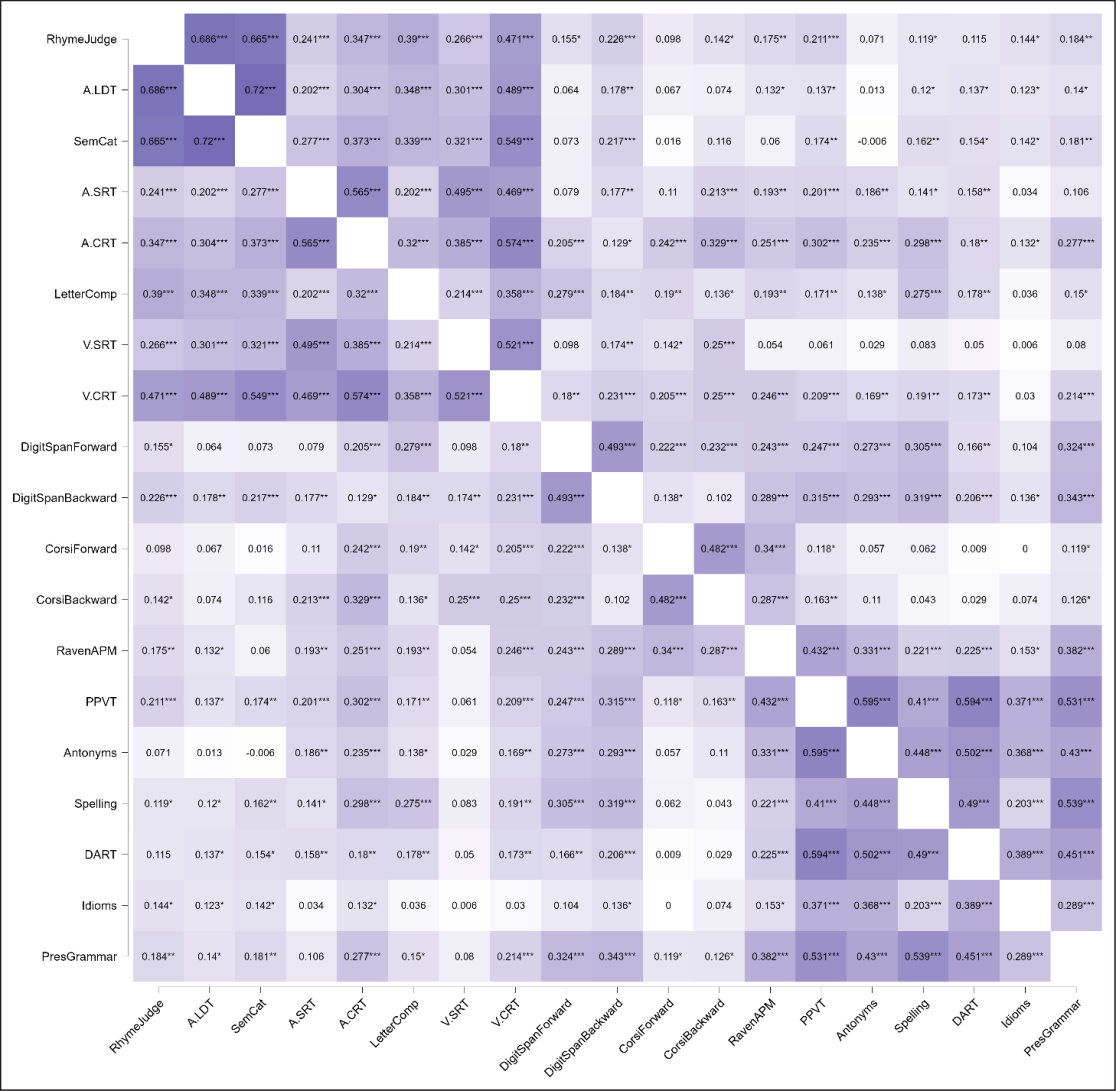
Pearson’s correlations between all 19 scores, represented as heatmap. Darker colours represent stronger correlations and lighter colours represent weaker correlations.

As a first analysis step, we submitted the 19 test scores to a Pearson’s correlation analysis. The outcome of this analysis is plotted as a heatmap in Figure 1. Stronger correlations are depicted in darker colours and weaker correlations in lighter colours. With two exceptions (semantic categorisation × antonym production, DART × Corsi block forward), all scores were positively correlated. This finding is in line with the positive manifold (Spearman, 1904), which reflects that performance of individuals on different cognitive task is predictive of their performance on other cognitive tasks.

The upper left quadrant of the heatmap features many moderate to strong correlations. They concerned the relationships between the tests measuring word recognition and processing speed, respectively (r’s ranging between .20 and .72). Similarly, as to be expected, the analysis revealed a moderate correlation between forward and backward Digit Span scores (r = .49) and between forward and backward Corsi block scores (r = .48). The scores reflecting linguistic experience (PPVT, antonym production, idiom recognition, DART, spelling, and prescriptive grammar) were moderately to strongly correlated (r ranging between .20 and .60). Finally, non-verbal reasoning (as measured through Raven’s Advanced Progressive Matrices), was most strongly correlated with receptive vocabulary (PPVT) and correlated weakly to moderately with the other scores.

The rich pattern of correlations shows that the tests included in the present analysis drew upon multiple overlapping skills. As discussed above, we addressed this issue by conducting psychometric network analysis in JASP (version 0.16.4). The signed and weighted network was estimated using the EBICglasso function (Epskamp, 2015). Correlation method was set to ‘Auto’; sample size was set to maximum and missing values were excluded pairwise. The tuning parameter, which controls the level of sparsity in the model, was set to .25. The purpose of this parameter is to find the right balance between liberal and conservative network estimation, with the goal being to estimate a network that contains ‘true’ edges. When the tuning parameter is low (e.g., .1), only a few edges are removed, likely resulting in the retention of spurious edges; when the tuning parameter is high (e.g., .5), many edges are removed, likely resulting in the removal of true edges in addition to the removal of spurious edges (Epskamp & Fried, 2018). We therefore chose the middle ground between a very liberal and very conservative estimation. This setting resulted in 78 of 171 potential edges (i.e., unique shared variance between test scores). The overall sparsity of the network was estimated to be .54. We compared these values to those resulting from setting the tuning parameter to .5 and .1, respectively. Setting it to 0.1 did not change the results; setting the tuning parameter to 0.5 removed 2 edges and increased the network sparsity value to 0.56. Since lower sparsity values are preferred (Goswami et al., 2021), we left the tuning parameter at 0.25.

**Figure 2:**
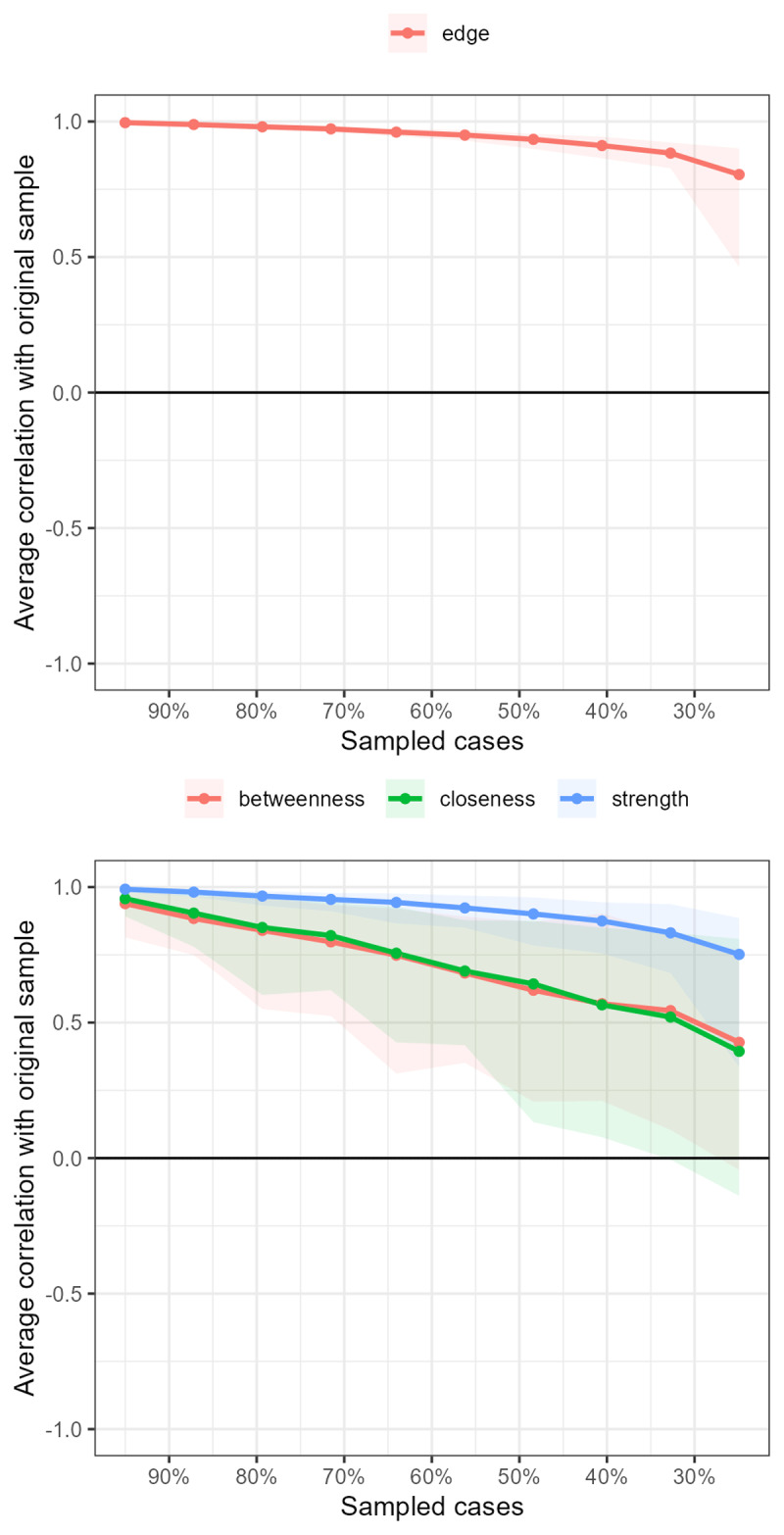
Outcome of network bootstrap procedure reflecting edge stability, betweenness, closeness, and strength.

Next, we assessed the stability of the network by bootstrapping it 1000 times. During this procedure, the network was re-estimated using different subsets of the original data (i.e., cases were systematically dropped) and was compared to the original network. Figure 2 shows the outcome of the bootstrapping procedure. As can be seen, in terms of edge stability, even when 70% of the cases were dropped, the average correlation with the original sample was still above .75, which suggests excellent stability. The same held true for the measure of strength (reflecting the degree to which each node is connected to other nodes in the network). In terms of betweenness (degree to which nodes stand between each other) and closeness (number of shortest paths between all nodes), however, the bootstrapping procedure revealed much less reliable results: When dropping around 40% of the original cases, the correlation with the original sample dropped below .7. The credible intervals suggest quite some variation. In our reporting and discussion of the results, we will therefore focus on the existence of links between nodes and their strength.

**Figure 3:**
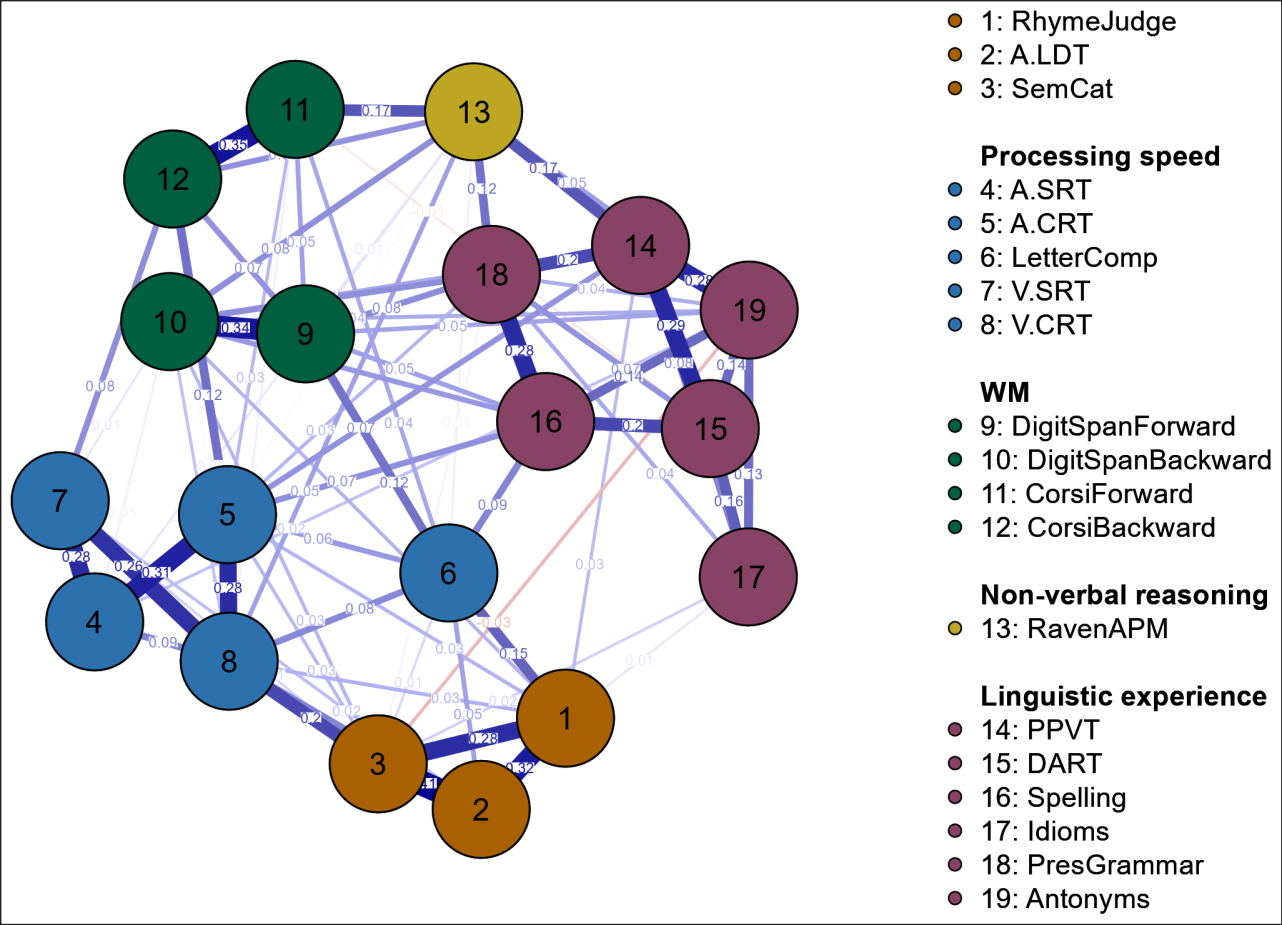
Psychometric network for the components of spoken word recognition. The strength of unique associations is represented by the thickness of the edges (i.e., the connections between the nodes). Distance between the nodes does not relate to the relationship between them.

We then plotted the network (Figure 3) such that positive edges would be depicted in blue and negative edges in red. To facilitate visual inspection, we plotted nodes reflecting the same psychological construct in the same colour. Note that the strength of unique associations is expressed in the edge thickness and the corresponding weight (Table 2).

**Table 2:** Network model weights matrix listing partial correlations between nodes.


VARIABLE	RJ	ALDT	SC	ASRT	ACRT	LC	VSRT	VCRT	DSF	DSB	CBF	CBB	RAPM	PPVT	DART	SP	IR	PG	AP

RJ	0.000	**0.322**	**0.275**	0.000	0.031	0.148	0.000	0.033	0.000	0.031	0.000	0.000	0.000	0.026	0.000	0.000	0.009	0.000	0.000

ALDT	**0.322**	0.000	**0.414**	0.000	0.000	0.051	0.024	0.089	0.000	0.000	0.000	0.000	0.000	0.000	0.000	0.000	0.000	0.000	0.000

SC	**0.275**	**0.414**	0.000	0.000	0.027	0.013	0.014	0.201	0.000	0.029	0.000	0.000	0.000	0.000	0.000	0.000	0.016	0.003	–0.028

ASRT	0.000	0.000	0.000	0.000	0.310	0.000	0.279	0.093	0.000	0.006	0.000	0.000	0.009	0.000	0.000	0.000	0.000	0.000	0.023

ACRT	**0.031**	0.000	**0.027**	0.310	0.000	0.062	0.000	0.277	0.000	0.000	0.028	0.123	0.001	0.067	0.000	0.074	0.000	0.029	0.000

LC	**0.148**	**0.051**	**0.013**	0.000	0.062	0.000	0.000	0.082	0.119	0.000	0.043	0.000	0.005	0.000	0.000	0.090	0.000	0.000	0.000

VSRT	0.000	**0.024**	**0.014**	0.279	0.000	0.000	0.000	0.262	0.000	0.009	0.000	0.075	0.000	0.000	0.000	0.000	0.000	0.000	0.000

VCRT	**0.033**	**0.089**	**0.201**	0.093	0.277	0.082	0.262	0.000	0.000	0.023	0.005	0.002	0.050	0.000	0.000	0.000	0.000	0.000	0.000

DSF	0.000	0.000	0.000	0.000	0.000	0.119	0.000	0.000	0.000	0.342	0.050	0.069	0.008	0.000	0.000	0.052	0.000	0.076	0.047

DSB	**0.031**	0.000	**0.029**	0.006	0.000	0.000	0.009	0.023	0.342	0.000	0.000	0.000	0.075	0.042	0.000	0.060	0.000	0.074	0.038

CBF	0.000	0.000	0.000	0.000	0.028	0.043	0.000	0.005	0.050	0.000	0.000	0.349	0.174	0.000	–0.008	0.000	0.000	0.000	0.000

CBB	0.000	0.000	0.000	0.000	0.123	0.000	0.075	0.002	0.069	0.000	0.349	0.000	0.070	0.000	0.000	0.000	0.000	0.000	0.000

RAPM	0.000	0.000	0.000	0.009	0.001	0.005	0.000	0.050	0.008	0.075	0.174	0.070	0.000	0.173	0.000	0.000	0.000	0.120	0.053

PPVT	**0.026**	0.000	0.000	0.000	0.067	0.000	0.000	0.000	0.000	0.042	0.000	0.000	0.173	0.000	0.288	0.000	0.084	0.200	0.283

DART	0.000	0.000	0.000	0.000	0.000	0.000	0.000	0.000	0.000	0.000	–0.008	0.000	0.000	0.288	0.000	0.204	0.155	0.068	0.138

SP	0.000	0.000	0.000	0.000	0.074	0.090	0.000	0.000	0.052	0.060	0.000	0.000	0.000	0.000	0.204	0.000	0.000	0.279	0.137

IR	**0.009**	0.000	**0.016**	0.000	0.000	0.000	0.000	0.000	0.000	0.000	0.000	0.000	0.000	0.084	0.155	0.000	0.000	0.040	0.128

PG	0.000	0.000	**0.003**	0.000	0.029	0.000	0.000	0.000	0.076	0.074	0.000	0.000	0.120	0.200	0.068	0.279	0.040	0.000	0.037

AP	0.000	0.000	**–0.028**	0.023	0.000	0.000	0.000	0.000	0.047	0.038	0.000	0.000	0.053	0.283	0.138	0.137	0.128	0.037	0.000


*Note*: RJ = Rhyme judgment, ALDT = Auditory lexical decision test, SC = Semantic categorisation, ASRT = Auditory simple reaction time, ACRT = Auditory choice reaction time, LC = Letter comparison, VSRT = Visual simple reaction time, VCRT = Visual choice reaction time, DSF = Digit span forward, DSB = Digit Span backward, CBF = Corsi block forward, CBB = Corsi block backward, RAPM = Raven’s Advanced Progressive Matrices, PPVT = Peabody Picture Vocabulary Test, DART = Dutch Author Recognition Test, SP = Spelling test, IR = Idiom recognition, PG = Prescriptive grammar, AP = Antonym production.

As mentioned earlier, the complete dataset consists of the results of two separate studies, conducted at different times. For the present report, we pooled the datasets in order to have sufficient power for conducting the network analysis. However, we also analysed the two sets separately. The networks are shown in Appendix A. As can be seen, apart from numerical differences in edge weights and two negative links (most likely spurious, due to the reduced sample size), the overall pattern of links was very similar across both networks (i.e., datasets). In the remainder of this text, we refer to the combined data set and discuss to what extent the results of the network analysis were consistent with each of our five hypotheses.

### (1) Links between nodes reflecting the same psychological construct

Consistent with the information gleaned from inspecting the matrix of correlations, we see that the tests designed to assess a common construct clustered together. This holds for the six linguistic experience tests, for the five tests of processing speed, and the three word recognition tests. The tests measuring auditory and visual working memory were correlated, but less strongly than one might expect. That is, the two auditory working memory tests, forward and backward Digit Span, were tightly linked, as were the two visual working memory tasks, forward and backward Corsi block tests. The links across the two pairs of tests were weaker, most likely because of the difference in test modality. Correlations of similar strength were reported in a study by Kessels et al. (2008). However, in general, the clustering of the nodes confirms the convergent validity of our tests.

Zooming in on the individual clusters, we see that the links varied in strength. In the linguistic experience cluster, for example, PPVT and DART (reflecting receptive vocabulary size and exposure to literary texts, respectively) were most strongly correlated with the other nodes. Idiom recognition, on the other hand, showed the weakest links to the other linguistic experience nodes. In the processing speed cluster, auditory and visual, simple and choice reaction time tests were strongly correlated. The letter comparison test showed the weakest links to the other tests. A similar pattern has been shown before (Hintz et al., 2022), and is likely due to the fact that letter Comparison is more complex than the other tests measuring processing speed. In the letter comparison test, participants are instructed to compare two strings of letters, presented one below the other, as quickly as possible. The task thus involves rapid visual processing and a speeded motor response, but *also*, to some extent, working memory as the first letter sequence must be kept active in memory for comparison with the second letter sequence. This explains the link between letter comparison and forward Digit Span.

### (2) Links between spoken word recognition nodes

Within the word recognition cluster, we see strong links between the three nodes. Critically, the semantic categorisation test was more strongly associated with the lexical decision test than with the rhyme judgment test. As discussed above, this pattern was expected on theoretical grounds, based on the assumptions underlying POT (Kovacs and Conway, 2016). POT assumes that the overlap in domain-specific and domain-general processes involved in a set of tasks (e.g., spoken word recognition) drives the strength of the correlations between these tasks. In terms of spoken word recognition, there is differential overlap in the representations that need to be activated for the three tests used here. Specifically, semantic categorisation is more similar to lexical decision than to rhyme judgment, since the latter requires processing at the sublexical level, but not at lexical and semantic levels, which must be accessed in the lexical decision and semantic categorisation tests. This account of the data is thus in line with the assumptions underlying POT and is generally in line with earlier research on spoken word recognition (Eisner & McQueen, 2018, for review). The main focus of the present work was, however, on the links between the nodes of spoken word recognition and those reflecting other verbal and non-verbal skills.

### (3) Links between spoken word recognition and non-verbal processing speed

Based on our earlier work (Hintz et al., 2020a), we predicted relationships between the tests measuring word recognition and those measuring processing speed. Our network analysis indeed revealed that the word recognition cluster (i.e., all three tasks) was linked to processing speed. The links indicate that both sets of tests draw upon shared underlying skills. In other words, participants’ response speed in the word recognition tests is governed by both domain-specific (e.g., processing at sublexical, lexical and semantic levels) and domain-general skills—the latter are also implicated in responding to non-verbal stimuli. It is important to highlight again that when calculating the link between a pair of nodes, variance shared with the rest of the network is partialled out. Since the five processing speed and three spoken word recognition tests *all* involved speeded button presses, the links between the word recognition and processing speed nodes are unlikely to simply reflect variance associated with carrying out a manual response.

All three word recognition tests were linked to the letter comparison and to the visual choice RT nodes. These links are interesting and suggest that variance is shared between these (visual) processing speed and the word recognition tests that is not shared with other tests in the network. One conjecture is that the links reflect the involvement of visual processes during spoken word recognition (e.g., the activation of orthographic representations, Pattamadilok et al., 2009; Taft et al., 2008) and that enhanced visual processing skills benefit spoken word recognition.

In terms of the number of links, the rhyme judgment node showed links to three of the five processing speed nodes, lexical decision to three, and semantic categorisation to four. Thus, the semantic categorisation test was the one that showed most overlap in unique variance with the individual processing speed tests. However, considering the links’ strength (Table 2), it becomes clear that the overlap was not very strong, which was the case for all tests of spoken word recognition: Partial correlation strength with processing speed tests ranged between .03 and .15 in the rhyme judgment, between .02 and .09 in the lexical decision and between .01 and .20 in the semantic categorisation test. Thus, overall, the pattern displayed in the network does not suggest a clear increase in the number or strength of links with processing speed as word recognition task complexity increases.

As discussed in the Introduction, this finding can be interpreted in multiple ways. One is that there is little variability pertaining to the speed with which activation cascades through the levels of representation in the word recognition system. Given the approximately two decades of experience in comprehending spoken words that our participants have had, mapping the incoming speech signal onto phonological, word form and semantic representations may have turned into an automatised process, which makes the strong involvement of domain-general processing speed unnecessary. One might observe different patterns in developing language users (see Bloom & Markson, 1998, for discussion). On the basis of the present data, one may thus conclude that the speed of recognising spoken words (and phoneme sequences) is predominantly driven by domain-specific processes and to a lesser extent by domain-general processing speed. This conclusion is supported by the fact that the links between the word recognition tests were much stronger than the links between the word recognition and the processing speed tests.

To complement the present psychometric network approach, future research could use diffusion modelling (e.g., Lerche et al., 2020; Voss et al., 2013), which uses mathematical models to segregate stimulus processing (referred to as ‘information accumulation’ or ‘drift rate’) from carrying out a speeded manual response that involves a choice component (‘non-decision time’), on the word recognition and processing speed tests. This type of analysis enables researchers to directly correlate the processes involved in stimulus processing (e.g., spoken words, tones, letter sequences) and the processes involved in carrying out a speeded response.

### (4) Links between spoken word recognition and linguistic experience

We hypothesised that spoken word recognition, in particular lexical decision and semantic categorisation, would show links to linguistic experience. Semantic categorisation showed links to three linguistic experience tests, idiom recognition, prescriptive grammar, and antonym production, however, the relationship with antonym production was negative. Moreover, rhyme judgement had links with the PPVT and with idiom recognition. The strength of all positive links ranged between .003 and .031 and was thus not very strong. The finding that there is apparently little communality in the skills implicated in the word recognition tests and in the linguistic experience tests may come as a surprise, as listeners must use their linguistic knowledge to carry out the tests, and listeners who do not know any Dutch will perform at chance in these tests. As discussed above, Diependaele et al. (2013) argued that enhanced linguistic experience benefits word recognition (speed), as individuals with more linguistic experience have sharper or more entrenched lexical representations than individuals with limited experience (see Huettig & Pickering, 2019, for a similar point). It is important to highlight that Diependaele et al.’s claim is based on an interaction between word frequency and receptive vocabulary size on a visual lexical decision task: Individuals with smaller vocabularies showed a larger RT difference between high- and low-frequency words compared to individuals with larger vocabularies. Diependaele and colleagues (2013) interpreted this finding as showing that the lexical representations in individuals with larger vocabularies (i.e., individuals with enhanced linguistic experience) are well developed (i.e., entrenched) for both high- and low-frequency words. A likely reason why we did not see such a relationship between the lexical decision task and linguistic experience relates to the inclusion of multiple constructs and multiple tests per construct in our network analysis. Diependaele et al. (2013) had assessed vocabulary size in their participants using a single measure for receptive vocabulary (LexTALE, Lemhöfer & Broersma, 2012). As can be seen in the Pearson’s correlation analysis (Figure 1), PPVT, spelling, idiom recognition and prescriptive grammar tests were all significantly correlated with all word recognition tests. However, these relationships most likely dropped below significance level when variance shared with other linguistic experience and non-verbal tests was accounted for. A complementary explanation could be that the sample of participants tested here, mostly university students, was too homogenous. Although these individuals differed in their linguistic experience, their lexical representations were likely well entrenched and featured little variability. It is possible that the inclusion of more participants with diverse educational backgrounds is required to observe robust effects of linguistic experience on the speed of word recognition.

### (5) Links between spoken word recognition and working memory

As predicted, we observed that rhyme judgment and semantic categorisation nodes were both linked to working memory (Digit Span). These relationships are most likely due to the nature of the tasks, which both involved keeping linguistic information active for a short period during the trial.

### Additional findings

Although not our main focus, the network analysis revealed that performance on the non-verbal reasoning test (Raven’s APM) was associated with tests of linguistic experience, most strongly PPVT, which taps vocabulary knowledge. This link is consistent with earlier findings by Simpson-Kent et al. (2020) and suggests that the PPVT involves a strong reasoning component. The auditory working memory scores were also linked to some of the linguistic experience scores (e.g., PPVT, spelling), which may suggest that good working memory skills benefit the acquisition of linguistic knowledge (e.g., word meanings and orthographic representations, Gathercole, 2006). Working memory was also linked to non-verbal reasoning, which is plausible as carrying out Raven’s APM requires the short-term maintenance of rules and dependencies for selecting the shape that completes the matrix of geometrical figures.

## Conclusions and Outlook

The present paper had two aims: to report novel findings about the relationship between the word recognition system and other components of the cognitive system, and to illustrate the use of network analysis in individual-differences work in psycholinguistics.

Turning to the first aim, we were specifically interested in the link between word recognition and processing speed. The network analysis revealed links between the two sets of tasks, implying that the spoken word recognition tasks recruited processes also involved in speeded responding to non-verbal stimuli. This suggests that the speed of access to words is partly determined by domain-general response speed but hinges, to a larger extent, on processes that are specific to retrieving lexical representations.

An important task for future research is to replicate these findings, particularly with broader groups of participants. In the present study, the unique links between scores were generally not strong (compared, for instance, to those seen in Goring et al., 2021). This may in part be due to the fact that most of our participants were university students or graduates. Stronger, and possibly different, links between scores may arise in assessments of participants with more varied educational backgrounds (see also Engelhardt et al., 2017). We were, for instance, surprised to see that linguistic experience had no obvious impact on the speed of word recognition. We would expect a different picture, with positive links between indicators of linguistic knowledge and word recognition speed, in a more heterogeneous sample.

Turning to the second aim, we illustrated how network analysis can be used in psycholinguistics. We find this tool appealing because researchers do not have to make a priori assumptions about latent variables determining behaviour. In particular, the analysis operates at the level of the test scores by computing *unique* links between them. These links are visualised in an intuitive way. Thus, the analysis does not require the computation of higher-order factors, which may or may not have a psychological and/or neurobiological substrate (Borsboom et al., 2003). This approach to data analysis fits well with the view that linguistic tasks, such as spoken word recognition, involve shared or overlapping sets of skills, and that, in general, participants’ scores in psycholinguistic and cognitive tests are correlated because the tests tap overlapping skills sets.

We think that the approach illustrated here can be fruitfully applied to many areas of psycholinguistics. In the present project, we studied the narrowly defined skill set of auditory word recognition. In ongoing work, we explore the links within a more varied set of linguistic tasks, including word and sentence comprehension and production, and the links of these tasks to domain-general skills. We hope that this work as well as work in other labs will lead to comprehensive theories about the ways the linguistic processing system is embedded in the broader cognitive system.

## Data Accessibility Statement

The pre-processed data used for the present analyses are available at the archive of the Max Planck Institute for Psycholinguistics at https://hdl.handle.net/1839/3b656615-6a9a-46d2-a68b-580151f27d95. The archive also contains the JASP analysis output.

## Additional File

The additional file for this article can be found as follows:

10.5334/joc.340.s1 Appendix A. Network analysis of Dataset A and B.
