# Supplementary material for: Using Psychometric Network Analysis to Examine the Components of Spoken Word Recognition
Source: J Cogn. 2024 Jan 10;7(1):10. doi: 10.5334/joc.340 (PMC10786093; doi:10.5334/joc.340)
Supplement: Appendix A. — Network analysis of Dataset A and B. [file joc-7-1-340-s1.pdf]

Using psychometric network analysis to examine the components of spoken word recognition

Florian Hintz[1,2], James M. McQueen[2,3], and Antje S. Meyer[2,3]

[1]Philipps University of Marburg, Marburg, Germany

[2]Max Planck Institute for Psycholinguistics, Nijmegen, The Netherlands

[3] Radboud University, Nijmegen, The Netherlands

Appendix A

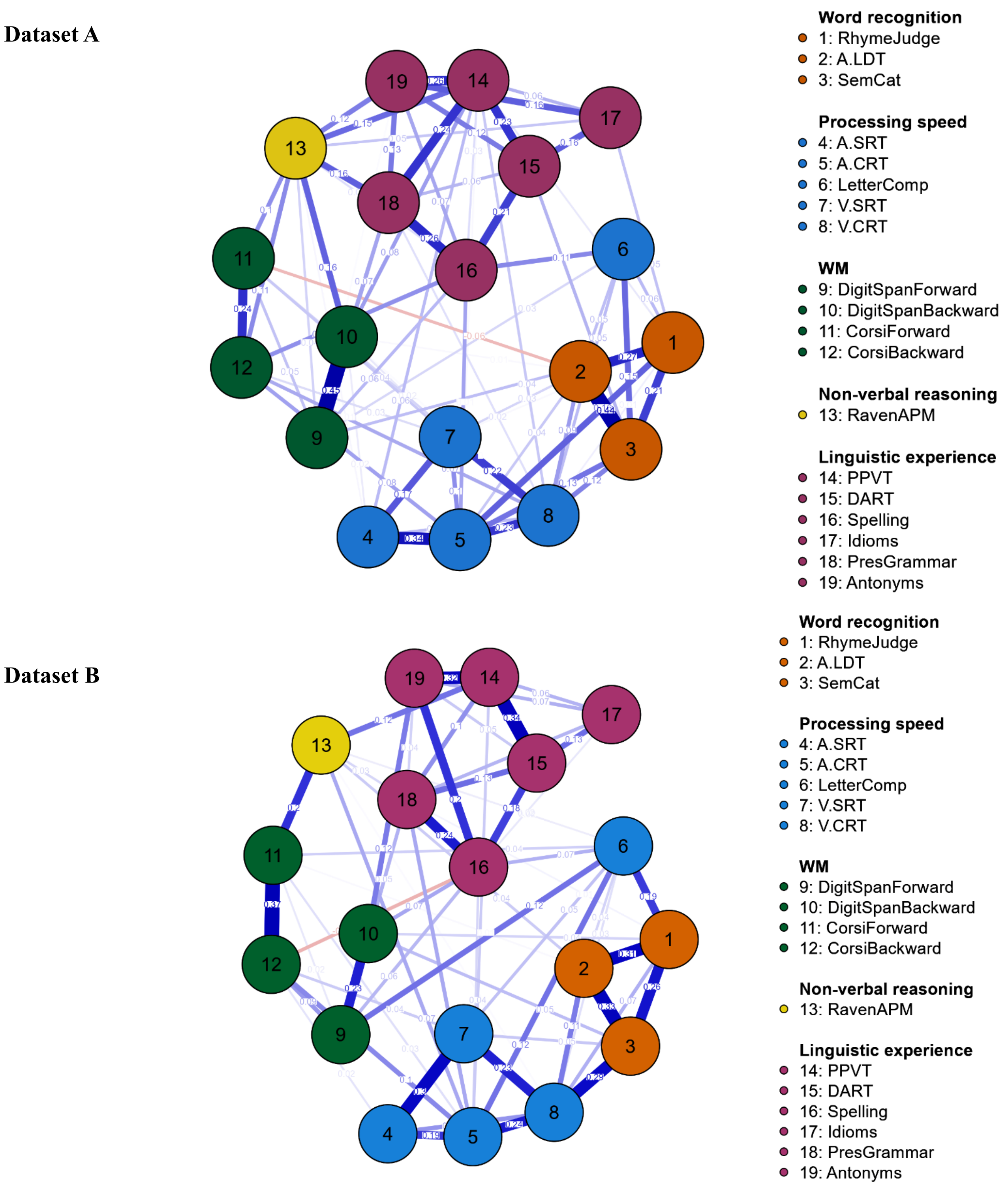

Figure 1: Psychometric network plots split out by dataset. The strength of unique associations is represented by the thickness of the edges (i.e., the connections between the nodes). Distance between the nodes does not relate to the relationship between them.

Dataset A

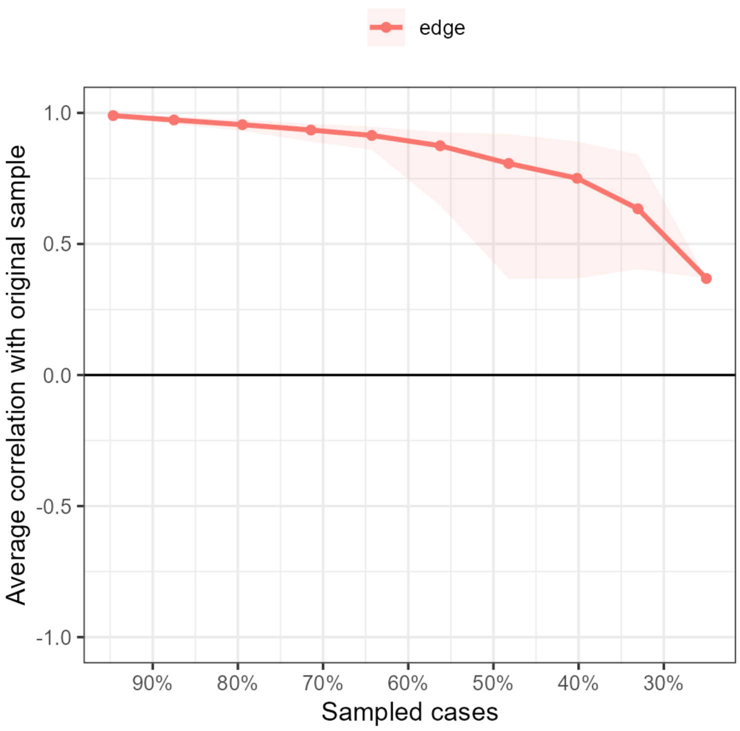

Dataset B

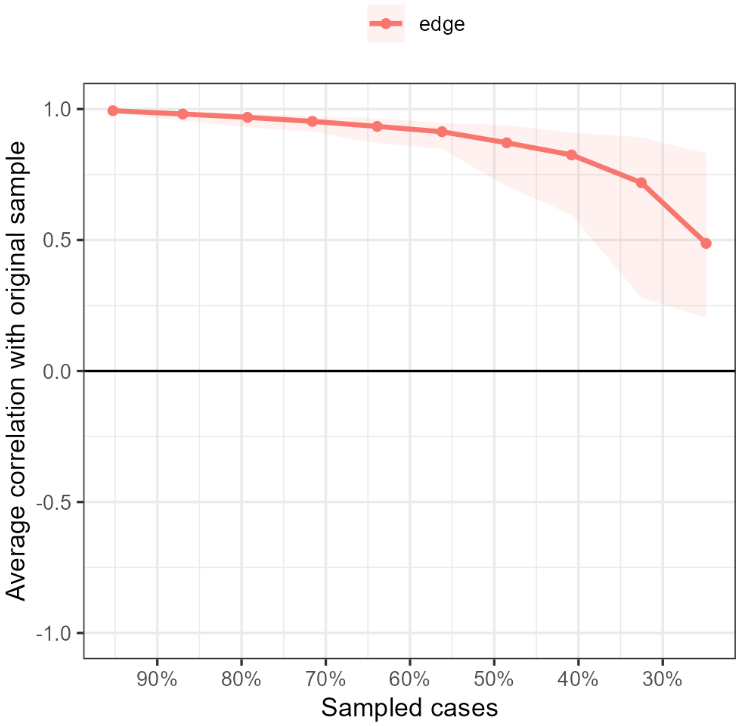

Figure 2: Outcome of network bootstrap procedure reflecting edge stability in Dataset A and B.

Dataset A

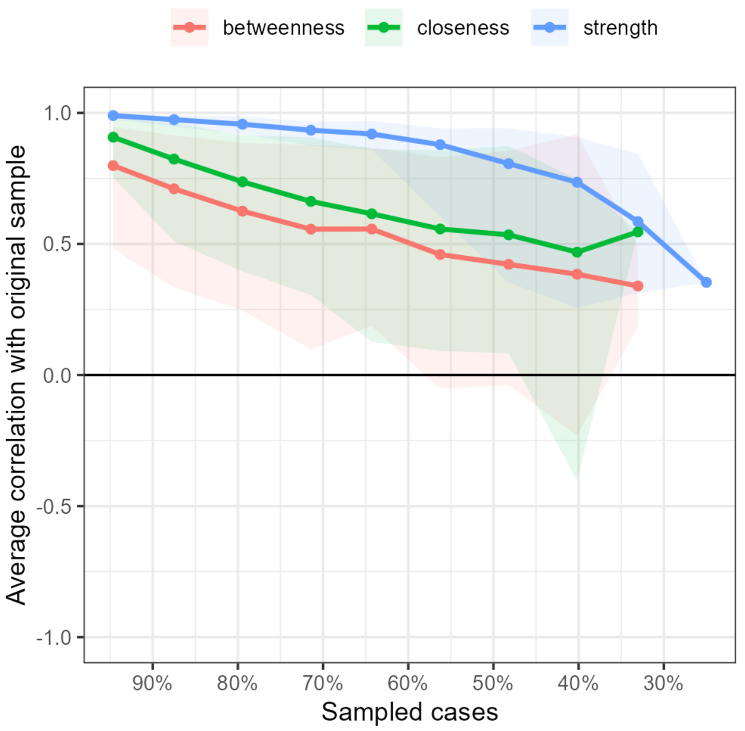

Dataset B

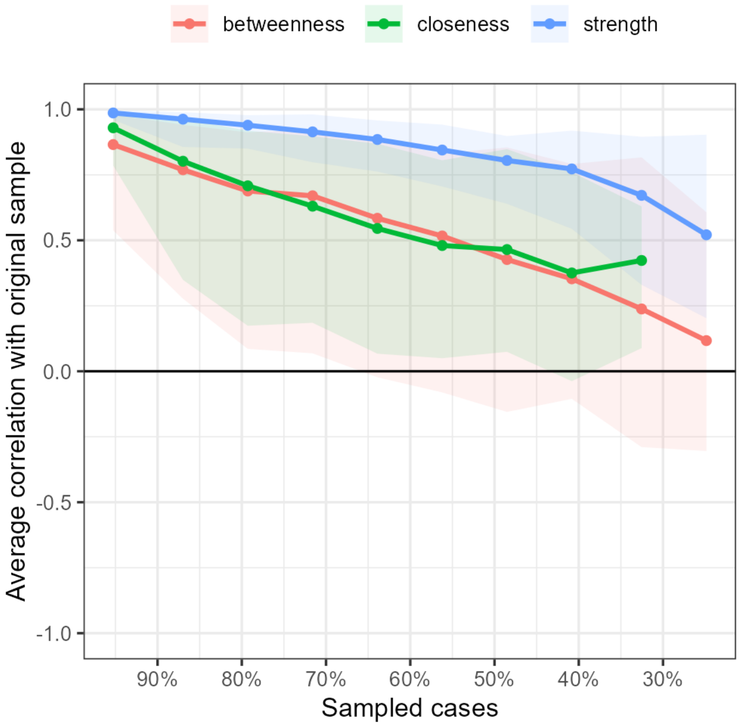

Figure 3: Outcome of network bootstrap procedure reflecting measures of centrality (i.e., betweenness, closeness, and strength) in Dataset A and B.

Table 1: Network model weights matrix listing partial correlations between nodes in *Dataset A*. Scores measuring the same psychological construct have a frame around them.

Variable	RJ	ALDT	SC	ASRT	ACRT	LC	VSRT	VCRT	DSF	DSB	CBF	CBB	RAPM	PPVT	DART	SP	IR	PG	AP
RJ	0.000	**0.270**	**0.211**	0.000	0.158	0.058	0.006	0.012	0.053	0.000	0.000	0.000	0.000	0.018	0.000	0.000	0.049	0.000	0.000
ALDT	**0.270**	0.000	**0.442**	0.000	0.044	0.054	0.034	0.086	0.000	0.006	-0.056	0.000	0.000	0.000	0.000	0.000	0.000	0.000	0.000
SC	**0.211**	**0.442**	0.000	0.000	0.133	0.147	0.000	0.115	0.000	0.000	0.000	0.000	0.000	0.000	0.062	0.000	0.000	0.000	0.000
ASRT	0.000	0.000	0.000	0.000	0.344	0.000	0.172	0.055	0.000	0.016	0.000	0.000	0.007	0.056	0.000	0.000	0.000	0.000	0.000
ACRT	**0.158**	**0.044**	**0.133**	0.344	0.000	0.000	0.101	0.233	0.000	0.000	0.000	0.080	0.000	0.006	0.000	0.078	0.000	0.000	0.000
LC	**0.058**	**0.054**	**0.147**	0.000	0.000	0.000	0.000	0.055	0.033	0.000	0.000	0.000	0.000	0.000	0.000	0.107	0.000	0.000	0.000
VSRT	**0.006**	**0.034**	0.000	0.172	0.101	0.000	0.000	0.216	0.000	0.018	0.045	0.027	0.000	0.000	0.000	0.000	0.000	0.000	0.000
VCRT	**0.012**	**0.086**	**0.115**	0.055	0.233	0.055	0.216	0.000	0.000	0.004	0.034	0.072	0.000	0.044	0.000	0.000	0.000	0.024	0.000
DSF	**0.053**	0.000	0.000	0.000	0.000	0.033	0.000	0.000	0.000	0.452	0.049	0.000	0.036	0.000	0.000	0.058	0.000	0.077	0.003
DSB	0.000	**0.006**	0.000	0.016	0.000	0.000	0.018	0.004	0.452	0.000	0.000	0.000	0.156	0.083	0.000	0.000	0.000	0.072	0.000
CBF	0.000	**-0.056**	0.000	0.000	0.000	0.000	0.045	0.034	0.049	0.000	0.000	0.244	0.098	0.000	0.000	0.000	0.000	0.000	0.000
CBB	0.000	0.000	0.000	0.000	0.080	0.000	0.027	0.072	0.000	0.000	0.244	0.000	0.105	0.000	0.000	0.097	0.000	0.000	0.000
RAPM	0.000	0.000	0.000	0.007	0.000	0.000	0.000	0.000	0.036	0.156	0.098	0.105	0.000	0.145	0.000	0.026	0.045	0.162	0.121
PPVT	**0.018**	0.000	0.000	0.056	0.006	0.000	0.000	0.044	0.000	0.083	0.000	0.000	0.145	0.000	0.231	0.026	0.063	0.236	0.259
DART	0.000	0.000	**0.062**	0.000	0.000	0.000	0.000	0.000	0.000	0.000	0.000	0.000	0.000	0.231	0.000	0.211	0.162	0.060	0.124
SP	0.000	0.000	0.000	0.000	0.078	0.107	0.000	0.000	0.058	0.000	0.000	0.097	0.026	0.026	0.211	0.000	0.000	0.263	0.074
IR	**0.049**	0.000	0.000	0.000	0.000	0.000	0.000	0.000	0.000	0.000	0.000	0.000	0.045	0.063	0.162	0.000	0.000	0.000	0.164
PG	0.000	0.000	0.000	0.000	0.000	0.000	0.000	0.024	0.077	0.072	0.000	0.000	0.162	0.236	0.060	0.263	0.000	0.000	0.126
AP	0.000	0.000	0.000	0.000	0.000	0.000	0.000	0.000	0.003	0.000	0.000	0.000	0.121	0.259	0.124	0.074	0.164	0.126	0.000

Note. RJ = Rhyme judgment, ALDT = Auditory lexical decision test, SC = Semantic categorisation, ASRT = Auditory simple reaction time, ACRT = Auditory choice reaction time, LC = Letter comparison, VSRT = Visual simple reaction time, VCRT = Visual choice reaction time, DSF = Digit span forward, DSB = Digit span backward, CBF = Corsi block clicking forward, CBB = Corsi block clicking backward, RAPM = Raven's Advanced Progressive Matrices, PPVT = Peabody Picture Vocabulary Test, DART = Dutch Author Recognition Test, SP = Spelling test, IR = Idiom recognition, PG = Prescriptive grammar, AP = Antonym production.

Table 2: Network model weights matrix listing partial correlations between nodes in *Dataset B*. Scores measuring the same psychological construct have a frame around them.

Variable	RJ	ALDT	SC	ASRT	ACRT	LC	VSRT	VCRT	DSF	DSB	CBF	CBB	RAPM	PPVT	DART	SP	IR	PG	AP
RJ	0.000	**0.313**	**0.257**	0.000	0.000	0.185	0.000	0.066	0.000	0.037	0.000	0.000	0.000	0.000	0.000	0.000	0.000	0.010	0.000
ALDT	**0.313**	0.000	**0.333**	0.000	0.000	0.036	0.000	0.114	0.000	0.000	0.006	0.000	0.044	0.000	0.000	0.000	0.000	0.000	0.000
SC	**0.257**	**0.333**	0.000	0.000	0.000	0.000	0.047	0.287	0.000	0.055	0.000	0.000	0.000	0.000	0.000	0.000	0.000	0.000	0.000
ASRT	0.000	0.000	0.000	0.000	0.192	0.000	0.305	0.090	0.000	0.000	0.000	0.022	0.000	0.000	0.000	0.000	0.000	0.000	0.000
ACRT	0.000	0.000	0.000	0.192	0.000	0.122	0.000	0.240	0.000	0.000	0.026	0.103	0.072	0.043	0.000	0.035	0.000	0.074	0.000
LC	**0.185**	**0.036**	0.000	0.000	0.122	0.000	0.052	0.026	0.124	0.000	0.039	0.000	0.016	0.000	0.000	0.065	0.000	0.000	0.000
VSRT	0.000	0.000	**0.047**	0.305	0.000	0.052	0.000	0.229	0.000	0.000	0.000	0.044	0.000	0.000	0.000	0.000	0.000	0.000	0.000
VCRT	**0.066**	**0.114**	**0.287**	0.090	0.240	0.026	0.229	0.000	0.000	0.000	0.000	0.000	0.004	0.000	0.000	0.000	0.000	0.000	0.000
DSF	0.000	0.000	0.000	0.000	0.000	0.124	0.000	0.000	0.000	0.228	0.019	0.088	0.000	0.000	0.000	0.060	0.000	0.065	0.000
DSB	**0.037**	0.000	**0.055**	0.000	0.000	0.000	0.000	0.000	0.228	0.000	0.000	0.000	0.000	0.000	0.000	0.075	0.000	0.046	0.115
CBF	0.000	**0.006**	0.000	0.000	0.026	0.039	0.000	0.000	0.019	0.000	0.000	0.372	0.198	0.000	0.000	0.000	0.000	0.000	0.000
CBB	0.000	0.000	0.000	0.022	0.103	0.000	0.044	0.000	0.088	0.000	0.372	0.000	0.000	0.000	0.000	-0.065	0.000	0.000	0.000
RAPM	0.000	**0.044**	0.000	0.000	0.072	0.016	0.000	0.004	0.000	0.000	0.198	0.000	0.000	0.123	0.000	0.000	0.000	0.033	0.000
PPVT	0.000	0.000	0.000	0.000	0.043	0.000	0.000	0.000	0.000	0.000	0.000	0.000	0.123	0.000	0.340	0.000	0.063	0.095	0.315
DART	0.000	0.000	0.000	0.000	0.000	0.000	0.000	0.000	0.000	0.000	0.000	0.000	0.000	0.340	0.000	0.178	0.128	0.128	0.045
SP	0.000	0.000	0.000	0.000	0.035	0.065	0.000	0.000	0.060	0.075	0.000	-0.065	0.000	0.000	0.178	0.000	0.019	0.237	0.197
IR	0.000	0.000	0.000	0.000	0.000	0.000	0.000	0.000	0.000	0.000	0.000	0.000	0.000	0.063	0.128	0.019	0.000	0.063	0.067
PG	**0.010**	0.000	0.000	0.000	0.074	0.000	0.000	0.000	0.065	0.046	0.000	0.000	0.033	0.095	0.128	0.237	0.063	0.000	0.038
AP	0.000	0.000	0.000	0.000	0.000	0.000	0.000	0.000	0.000	0.115	0.000	0.000	0.000	0.315	0.045	0.197	0.067	0.038	0.000

Note. RJ = Rhyme judgment, ALDT = Auditory lexical decision test, SC = Semantic categorisation, ASRT = Auditory simple reaction time, ACRT = Auditory choice reaction time, LC = Letter comparison, VSRT = Visual simple reaction time, VCRT = Visual choice reaction time, DSF = Digit span forward, DSB = Digit span backward, CBF = Corsi block clicking forward, CBB = Corsi block clicking backward, RAPM = Raven's Advanced Progressive Matrices, PPVT = Peabody Picture Vocabulary Test, DART = Dutch Author Recognition Test, SP = Spelling test, IR = Idiom recognition, PG = Prescriptive grammar, AP = Antonym production.

In a supplementary analysis suggested by an anonymous reviewer, we explored the individual network structures of Dataset A (Hintz et al., 2020b) and Dataset B (Hintz et al., in prep.). As the main analysis, the supplementary analysis was conducted in JASP (version 0.16.4). The settings were the same as for the main analysis: The signed and weighted networks were estimated using the EBICglasso function (Epskamp, 2015). Correlation method was set to 'Auto'; sample size was set to maximum and missing values were excluded pairwise. The tuning parameter, which controls the level of sparsity in the model, was set to .25. We also assessed the stability of each network by bootstrapping it 1000 times. The results of the supplementary analysis are presented in Figure 1 (network plots), Figure 2 (edge stability), Figure 3 (closeness, betweenness, strength) and in Tables 1 and 2 (edge weights).

As to be expected, the edge stability (Figure 2) was slightly better for Dataset B (n = 169) than for Dataset A (n = 112) due to the larger sample size. The bootstrapped measures of centrality were similar across the datasets: Whereas 'strength' in both datasets still showed a correlation of .75 with the original sample even when 60% of the cases were dropped, the measures of betweenness (degree to which nodes stand between each other) and closeness (number of shortest paths between all nodes) were quite unreliable.

Except for numerical differences in edge/link weights, the overall network structure was very similar across both datasets. For example, in both datasets we saw that auditory lexical decision and semantic categorization scores were more strongly linked than rhyme judgment was linked with each of these two nodes. Similarly, we observed that the scores reflecting linguistic experience clustered together in both networks. Interestingly, the link between spelling and antonym production scores was stronger in the Dataset B than in Dataset A. As in the main analysis, we observed that there were substantial links between scores stemming from the same working memory test (i.e., between digit span forward and backward and between Corsi block clicking forward and backward) but weaker links across modalities. Finally, in both networks we observed that simple and choice auditory and visual reaction time tasks clustered together and that

the node representing the letter comparison score was placed further apart from the other processing speed tests—more closely to the word recognition scores.

We observed two weak negative links: In Dataset A, Corsi forward was negatively linked with auditory lexical decision. In Dataset B, Corsi backward was negatively linked with the spelling node. Given the overall smaller sample size (compared to the main analysis) and the sample size difference across both datasets (Dataset B contained one third more participants than Dataset A), these links are hard to interpret. We note, however, that the overall pattern of links was very similar across both networks (i.e., datasets).

References

Hintz, F., Dijkhuis, M., van ‘t Hoff, V., McQueen, J. M., & Meyer, A. S. (2020b). A behavioural dataset for studying individual differences in language skills. Scientific Data, 7(1), 429.

Hintz, F., Kievit, R. A., McQueen, J. M., & Meyer, A. S. (in prep.). Assessing the principal dimensions of speaking and listening skills.
